# The neural correlates of in-group and self-face perception: is there overlap for high identifiers?

**DOI:** 10.3389/fnhum.2013.00528

**Published:** 2013-09-03

**Authors:** Daan Scheepers, Belle Derks, Sander Nieuwenhuis, Gert-Jan Lelieveld, Félice Van Nunspeet, Serge A. R. B. Rombouts, Mischa de Rover

**Affiliations:** ^1^Department of Psychology, Leiden UniversityLeiden, Netherlands; ^2^Leiden Institute for Brain and CognitionLeiden, Netherlands; ^3^Department of Radiology, Leiden University Medical CentreLeiden, Netherlands

**Keywords:** social identity, functional neuroimaging, self-perception, group identification

## Abstract

Social identity, the part of the self-concept derived from group membership, is a key explanatory construct for a wide variety of behaviors, ranging from organizational commitment to discrimination toward out-groups. Using functional magnetic resonance imaging (fMRI), we examined the neural basis of social identity through a comparison with the neural correlates of self-face perception. Participants viewed a series of pictures, one at a time, of themselves, a familiar other, in-group members, and out-group members. We created a contrast for self-face perception by subtracting brain activation in response to the familiar other from brain activation in response to the self face, and a contrast for social identity by subtracting brain activation in response to out-group faces from brain activation in response to in-group faces. In line with previous research, for the self—familiar other contrast we found activation in several right-hemisphere regions (inferior frontal gyrus, inferior and superior parietal lobules). In addition, we found activation in closely-adjacent brain areas for the social identity contrast. Importantly, significant clusters of activation in this in-group—out-group contrast only emerged to the extent that participants reported high identification with the in-group. These results suggest that self-perception and social identity depend on partly similar neural processes.

Social identity theory maintains that a person's self-concept consists of two parts: The personal self and the social self (Tajfel and Turner, 1979; Ellemers and Haslam, 2012). The social self, or “social identity,” is the part of identity derived from group membership (e.g., as “Female,” a “Red Sox-fan,” a “European,” a “Lefthander,” or “Catholic,” Tajfel and Turner, 1979). Social identity is a key explanatory construct for a wide variety of group behaviors, ranging from organizational commitment to discrimination against out-groups (Ellemers and Haslam, 2012). More generally, social identification with groups serves core human needs for belonging, social meaning, and self-esteem, and has important health consequences as it can form a buffer against pain and stress (Branscombe et al., 1999; Spears et al., 2004; Brewer, 2007; Hogg, 2009; Fiske, 2010; Jetten et al., 2011).

At the conceptual level, social identity has been described in terms of overlapping mental representations of self and in-group (Smith and Henry, 1996; Tropp and Wright, 2001; Otten and Epstude, 2006; Swann et al., 2009). In the current research we tested a novel prediction derived from this view, namely that when people see in-group faces, this activates similar brain areas as when people see their own face. Assuming that the personal self and social identity rely partly on comparable processes (Devos and Banaji, 2003), we hypothesized that similar brain areas are involved in defining and perceiving the self at the personal and the group level. However, because there are substantial differences in the extent to which people derive part of their identity from a particular group membership, we expect the activation of self-relevant brain areas in response to in-group faces to emerge as a function of the extent to which the person *identifies* with the in-group.

## The neural basis of visual self-face perception

In the past two decades, researchers have started to examine the neural basis of self-perception and self-awareness (see Lieberman, 2007, for an overview). An important strand of research within this area has focused on the neural correlates of self-face perception (Uddin et al., 2007; Platek et al., 2008; Devue and Brédart, 2011). In this research, participants typically view pictures of their own face and faces of familiar others (e.g., a friend or spouse) while brain activation is assessed using functional magnetic resonance imaging (fMRI). Compared to pictures of familiar others, pictures of the self typically activate a bilateral, but right-dominant, network (Keenan et al., 2001; Platek et al., 2004; Uddin et al., 2005; Lieberman, 2007; Sui and Han, 2007; see Platek et al., 2008, for meta-analytic evidence).

A recent overview of the literature (Devue and Brédart, 2011) indicated two regions of the right hemisphere that have been most frequently reported in fMRI research on self-face perception: The right inferior frontal gyrus and a right parietal network including the inferior and superior parietal lobule. This latter area seems to be particularly important for the perception of the self as a distinct entity (Uddin et al., 2005, 2007). Indeed, repetitive transcranial magnetic stimulation (rTMS) evidence indicates that a “virtual lesion” in the right (but not the left) inferior parietal lobule disrupts the ability to make distinctions between self and other (Uddin et al., 2006).

## Social identity and its neural substrates

Just as making distinctions between “self” and “other” (personal distinctiveness) forms the basis of the personal self, making distinctions between “us” and “them” (inter-group distinctiveness) forms the basis of social identity. More specifically, social identity theory is based on three principles: social categorization, social comparison, and social identity (Tajfel and Turner, 1979; Ellemers and Haslam, 2012). The theory starts with the notion that people categorize themselves and others as in-group and out-group members., In-group membership derives social meaning via social comparison of the in-group with the out-group, for example as a function of the social status of the group. This then yields “social identity,” namely the knowledge of group membership as a part of self, and the value and emotional significance attached to this (Tajfel and Turner, 1979). Thus, similar to the personal self, social identity contains both descriptive and evaluative aspects. The main difference between the personal self and social identity is that the social identity forms a higher, more inclusive, level of self-definition (Turner et al., 1987). Social-cognitive research has confirmed that similar principles underlie both levels of self-definition (Devos and Banaji, 2003; Volz et al., 2009).

As indicated above, while a sense of personal distinctiveness forms an important basis for a sense of (personal) self, group distinctiveness forms the basis for social identity. However, whereas personal distinctiveness is inherently present in healthy individuals by means of bodily distinctiveness from others, there is substantial variation in the extent to which people see the in-group as a clearly distinct entity (Spears et al., 2002). For example, while some left-handers see their in-group as clearly different from the out-group (right-handers), other left-handers do not have such a differentiated view of their in-group (e.g., in terms of the broader personality and other characteristics of its members), and see substantial overlap with the out-group. The tendency to have a clearly differentiated view of the in-group (vis-à-vis the out-group) is a direct function of the degree to which people *identify* with the group (Castano et al., 2002; Spears et al., 2004). That is, group identification forms the bridge between the in-group and the self (Tajfel and Turner, 1979; Deaux, 1996; Ellemers et al., 1999). Therefore, we expect that only people who identify strongly with an in-group activate similar brain areas when they see in-group faces and when they see their own face.

Previous neuroscience research has examined the affective and behavioral *consequences* of social identity, for example how the neural reward system responds to winning and losing an inter-group competition (Cikara et al., 2011). Moreover, research has also examined the neural substrates of in-group bias (i.e., the tendency to see one's group in a particularly positive light), a phenomenon that is closely related to the establishment of a positive social identity (Van Bavel et al., 2008; Volz et al., 2009). Research in the latter area has mainly focused on activation of the amygdala, which signals the motivational relevance of a stimulus. Research has indeed shown greater amygdala activation to in-group than to out-group faces (Van Bavel et al., 2008; Wright et al., 2008; cf. Hart et al., 2000).

Previous research has also examined the influence of racial identification (Mathur et al., 2012) and group identification (Molenberghs and Morrison, 2013) on neural responses to in-group and out-group stimuli. Mathur et al. report a relationship between racial identification and the activation of the “default network” (which is implied in self-reflection) when viewing in-group (as opposed to out-group) pictures. Moreover, in a study involving inter-group competition between two artificially-created groups (“minimal groups”), Molenberghs and Morrison found a relationship between group identification and activation of the medial prefrontal cortex, an area that has been implied in a variety of socio-cognitive processes, including the self (Lieberman, 2007). In the current work we build on this previous research, but make a direct comparison between neural activity related to the perception of the self-face, and neural activity related to the perception of in-group faces.

## The current research

As a starting point in the current research, we adapted a paradigm from research on self-face perception (Platek et al., 2004; Uddin et al., 2005), which we adapted by adding an inter-group dimension. While in the scanner, participants viewed pictures of themselves, a familiar other (friend), in-group members (students from Leiden University) and out-group members (students from the VU University Amsterdam). We expected that participants would differ in the strength of identification with their own university. Furthermore, by using university affiliation as categorization criterion rather than highly visible categories such as ethnicity, gender, or age, we could control for the physical characteristics of in-group and out-group targets. In the year the research was conducted (2011) the two universities were also quite close to each other in the Times Higher Education World University Ranking (Leiden: 54.4 points, 124th position; VU: 52.3 points, 139th position). The universities are also quite similar in size (Leiden: 19.000 students; VU: 18.000 students) and study profile, having strengths in similar areas of research and teaching (natural sciences, social sciences, humanities, medicine, law). In summary, the current context was ideal for studying basic social identity processes in a still realistic inter-group context.

## Method

### Participants

Forty-one right-handed male Leiden University students (*M*_age_ = 21) participated in return for €20. Participants provided informed consent according to the ethical guidelines of the Leiden University Medical Center.

### Stimuli

Participants viewed grayscale images of their own face (presented as in a picture), the face of a familiar other male student, and faces of 14 Caucasian male students (7 in-group, 7 out-group). All faces bore a neutral expression and eyes were directed at the camera. Based on a pretest (*N* = 47) of a larger sample of 36 pictures that were rated on attractiveness, we selected 14 pictures of average attractiveness. The assignment of pictures to the in- and out-group was pseudo-randomized over participants. Before the experiment, we checked whether participants were unfamiliar with in-group and out-group faces and replaced pictures of familiar faces with unfamiliar ones.

### Procedure

The study consisted of two sessions. In the first session participants were asked to bring a male Leiden student that they knew reasonably well^1^. We took pictures of both students and measured their identification with Leiden University using five items (e.g., “I feel a bond with Leiden students”; “I have a lot in common with other Leiden students”; α = 0.85).

**Figure 1:**
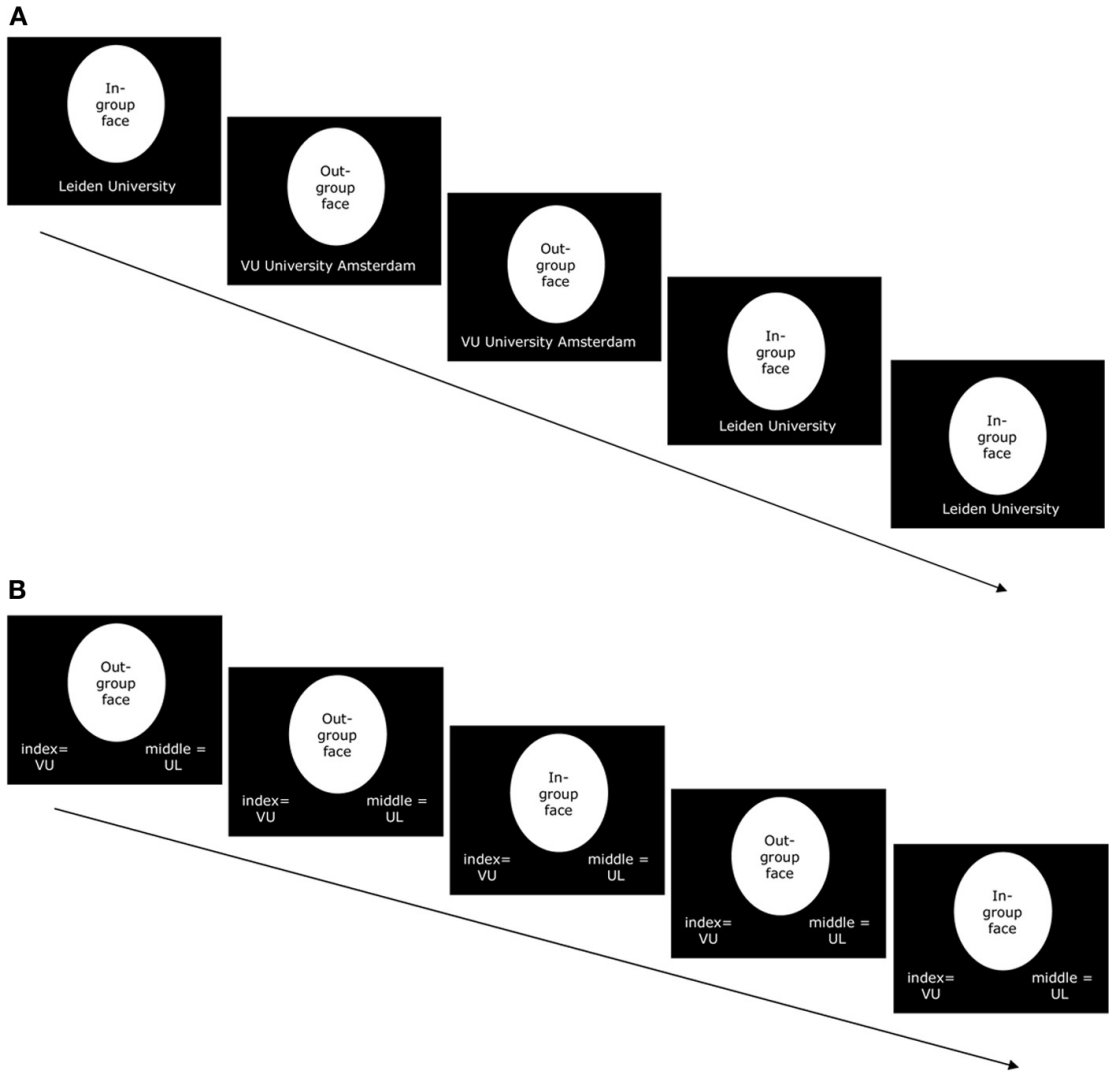
**(A)** Example of five screens in the presentation phase of the learning task that was performed outside of the scanner. Participants passively viewed and were instructed to memorize targets of their in-group (7 males Leiden University students) and their out-group (7 males VU University Amsterdam students). The university affiliation of each target appeared at the bottom of the screen. Screens appeared in complete sequences of all 14 targets (targets were presented in random order within each sequence). This sequence was presented 5 times. **(B)** Example of five trials in the testing phase of the learning task that was performed outside of the scanner. Participants were presented with targets of their in-group (7 males Leiden University students) or of their out-group (7 males VU University Amsterdam students) and had to indicate the university affiliation of each target (VU = VU University Amsterdam; UL = Leiden University). Trials appeared in complete sequences of all 14 targets (targets were presented in random order within each sequence). The testing phase ended when participants had flawlessly categorized all 14 targets in two subsequent sequences.

In a second session, before they went into the scanner, participants were asked to memorize the university affiliation of fourteen, as yet unfamiliar, male students: 7 in-group members and 7 out-group members. In the presentation phase the pictures of these 14 students were presented in random order on a computer screen; below each picture the university affiliation of the person was indicated (see Figure 1A). The sequence of 14 pictures was presented five times. After viewing the pictures, participants were tested on their ability to classify the pictures according to university affiliation. In this testing phase, all 14 pictures were again presented randomly in sequence, but this time without university labels (see Figure 1B). The participant's task was to indicate the university affiliation of the person on the picture, with the index and middle fingers of his right hand making use of two keys on the keyboard (counterbalanced across participants). After each response the participant received feedback about the correctness of his response. To make sure that every participant would be able to categorize the previously unfamiliar students during the subsequent, critical phase of the study, we set a fixed learning criterion: The complete sequence of 14 pictures had to be flawlessly categorized twice. Again, the stimuli were presented in series of 14 (comprising all stimuli). After a mistake, the sequence was first completed after which a new one started. This procedure was repeated until the participant completed two subsequent series correctly, after which he proceeded to the next phase, which took place in the scanner.

**Figure 2:**
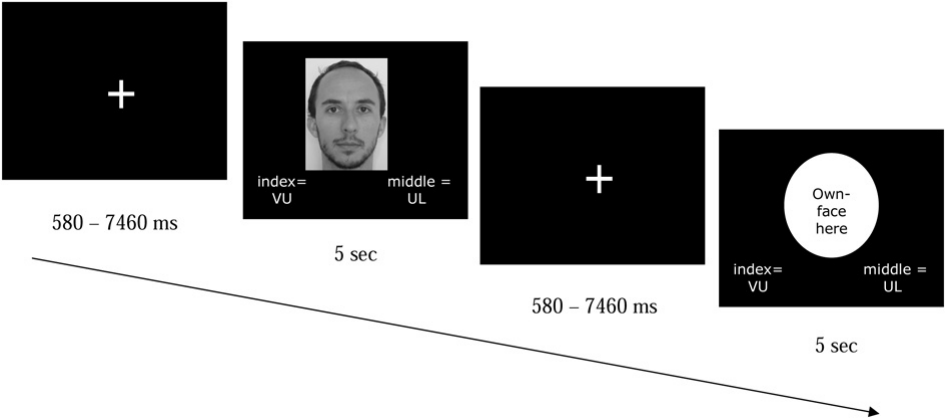
**Example of two trials of the task participants performed in the scanner**. Participants were presented with a picture of themselves, a familiar other, an ingroup or outgroup member. Although the group names appeared at the bottom of the screen for all picture types (VU = VU University Amsterdam; UL = Leiden University), participants' task was to respond only when a picture of an ingroup or outgroup members was presented. Target pictures stayed on the screen for 5 s, even after participants had responded.

Once in the scanner, participants were presented with the picture of themselves and their familiar other, and with the pictures of in-group targets and out-group targets, in random order (see Figure 2). They were asked to indicate the university affiliation of the in-group (UL) and out-group (VU) targets by pressing a corresponding button with the middle or index finger (counterbalanced across participants, but the same button-university combinations as in training phase) of their right hand. Although the response options were displayed along with each stimulus (see Figure 2), participants were explicitly asked not to respond to the pictures of themselves and their familiar other. During this phase, participants did not receive performance feedback.

We used an event-related design, in which stimuli were counterbalanced over 3 runs of 84 stimuli each. Per run each stimulus type (self, familiar other, in-group and out-group) was presented 21 times, for 5 s each time. Thus, each individual in-group/out-group target was presented three times per run, nine times in total. Pictures of self and familiar other were each presented 63 times across the experiment, so BOLD activity may have shown some habituation (Wedig et al., 2005). Stimuli were preceded by a fixation cross with a variable presentation time (580–7460 ms).

Participants were instructed to think about the person in the picture, for the full 5 s that the picture was shown. For in-group and out-group members, the participant was also instructed to indicate the person's university affiliation. After responding, the picture remained on the screen until the full 5 s had passed. These (verbally delivered) task instructions were repeated before the start of runs two and three.

As will be described in more detail in the next section, we operationalized self-face perception in terms of a contrast between self and familiar other (e.g., Uddin et al., 2005; Sui and Han, 2007). Social identity was operationalized in terms of a contrast between in-group and out-group, to which we added in-group identification as a covariate.

It should be noted that there are two important differences between the contrasts we created for self-face perception and social identity. First, in-group and out-group stimuli were explicitly categorized during the picture viewing task, while self and familiar other stimuli were not categorized. We required responses to in-group/out-group stimuli for two reasons: (1) To make sure participants kept on responding (e.g., they didn't fall asleep during a run); and (2) To be able to control for the number of errors participants made when categorizing in-group and out-group members in our analyses. Although we could safely assume that, especially in the absence of time pressure, people would immediately and correctly recognize pictures of themselves and their familiar other, we incorporated an additional check to verify that this was also the case for the (previously unfamiliar) in-group and out-group members. The motor activity associated with the responses to in-group and out-group stimuli (performed with adjacent fingers of the same hand) was essentially eliminated in the contrast comparing the in-group—out-group conditions. In addition, there may have emerged some neural activity associated with response inhibition in the self and familiar other conditions. That is, as indicated above, the possible response cues (left and right button options) from the in-group/out-group conditions were also displayed in the self and familiar other conditions, although the participant was explicitly instructed not to categorize these stimuli. Importantly, however, the inhibition of responses that may have been required when perceiving self and familiar other stimuli was also eliminated in the contrast comparing the self and familiar other conditions. Therefore, there was no confound in terms of motor activity or response inhibition between the contrasts for self-face perception and social identity.

The second, important, difference between the self-face perception and social identity contrasts concerns the addition of group identification as a covariate to the in-group—out-group contrast. As explained in the introduction, there is substantial variability in the extent to which people identify with, and thus derive part of their identity from, a particular group membership (not least because there are many different groups to which we belong, and many not out of choice). Note that this is in stark contrast to a sense of personal self, which is always to some extent present in healthy individuals. According to our rationale, in-group stimuli should only trigger social-identity-relevant brain responses to the extent that the person identifies with the group. Therefore, we took the variation in in-group identification into account when examining the social identity (i.e., in-group—out-group) contrast.

### MRI image acquisition and analyses

Images were collected with a 3-T Philips Achieva MRI scanner. Anatomical images were collected using a T1-weighted sequence (*TR* = 9.8 ms, *TE* = 4.59 ms, flip angle = 8°, 64 ^*^ 64 matrix, 140 slices, 0.875 × 0.875 × 1.2 mm). Visual stimuli were projected onto a screen that was viewed through a mirror at the head end of the magnet. Functional images were reconstructed from 38 transverse slices acquired using a T2^*^-weighted EPI sequence (*TR* = 2.2 s, *TE* = 30 ms, flip angle = 80°, 2.75 × 2.75 × 2.75 mm + 10% inter-slice gap). Image acquisition varied across trials with respect to stimulus onset, yielding an effectively higher temporal sampling rate. Three functional runs of 346 scans each were collected.

Data were preprocessed and analyzed with SPM8 (Wellcome Department of Cognitive Neurology, London). Functional images were motion-corrected using rigid-body realignment and then corrected for differences in timing of slice acquisition. The maximum amount of motion observed was 2 mm (in any direction). Each T1-weighted structural MR image was co-registered with the corresponding mean functional MR image and then segmented and spatially normalized to the Montreal Neurological Institute (MNI) reference brain template. Next, slice-timing- and motion-corrected functional images were normalized according to the same parameters and smoothed with a 8-mm full width at half-maximum Gaussian kernel.

For each participant, the blood oxygen-level dependent (BOLD) responses across the scanning run were modeled with a general linear model including four explanatory variables (box-car regressors with 5 s duration) that corresponded with the four experimental conditions: self, familiar other, in-group, and out-group. The explanatory variables were convolved with a canonical hemodynamic response function (HRF). In addition, the linear model included as regressors-of-no-interest session/subject-effects, errors (if any, *M* = 4.5, *SD* = 4.1, range 0–13), realignment parameters, and a temporal high-pass filter (1/128 Hz) to account for various low-frequency effects. For each voxel and each explanatory variable, a parameter estimate was generated that indicated the strength of covariance between the data and the HRF; these estimates were corrected for temporal autocorrelation using a first-order autoregressive model. Contrasts between parameter estimates for different conditions were calculated for each participant, and the results submitted to a group analysis that treated inter-subject variability as a random effect. In addition, for the group analyses of the in-group—out-group contrast, the participants' in-group identification scores were added as a covariate in the analysis. SPM8 automatically orthogonalized this parametric regressor with regard to the main trial regressor. Statistical parametric maps were derived from the resulting *t*-values associated with each voxel.

We analyzed the data in two steps. As a first step, we performed whole-brain analyses to search for self-related brain areas. Specifically, we sought to identify brain areas sensitive to self-face perception and areas sensitive to in-group—out-group distinction, as a function of group identification (social identity). Therefore, we examined the self—familiar other contrast and the in-group—out-group X identification contrast, with a threshold of *p* < 0.0005 (uncorrected) and a contiguity threshold of 20 voxels as a precaution against type-1 errors (Lieberman and Cunningham, 2009). To interpret the peaks of activation clusters we used the WFU Pickatlas (Maldjian et al., 2003, 2004). The self—familiar other and in-group—out-group X identification contrasts revealed that self- and in-group faces activated several closely adjacent areas in the right hemisphere. As a second step, we attempted to statistically support this impression. To this end we created spheres with an 8-mm radius around the peaks of activation clusters in the in-group—out-group X identification contrast. Then, we tested whether these regions contained voxel clusters that were more activated by self than by familiar other, using a small-volume correction (*p* < 0.05, cluster-corrected).

## Results

### Behavioral data

During the learning phase, group identification was negatively related to accuracy in categorizing in-group targets (*r* = −0.41, *p* = 0.008). This replicates previous research on in-group over-exclusion (Castano et al., 2002) and shows that those who identify relatively strongly with their group are more cautious when including people in their in-group, leading them to initially classify more targets as out-group-members. Importantly, identification was not related to the number of series participants needed to see to learn to categorize in-group and out-group stimuli flawlessly during the learning phase (*r* = 0.03); therefore, identification was unrelated to the frequency with which participants had seen the stimuli before going into the scanner.

**Figure 3:**
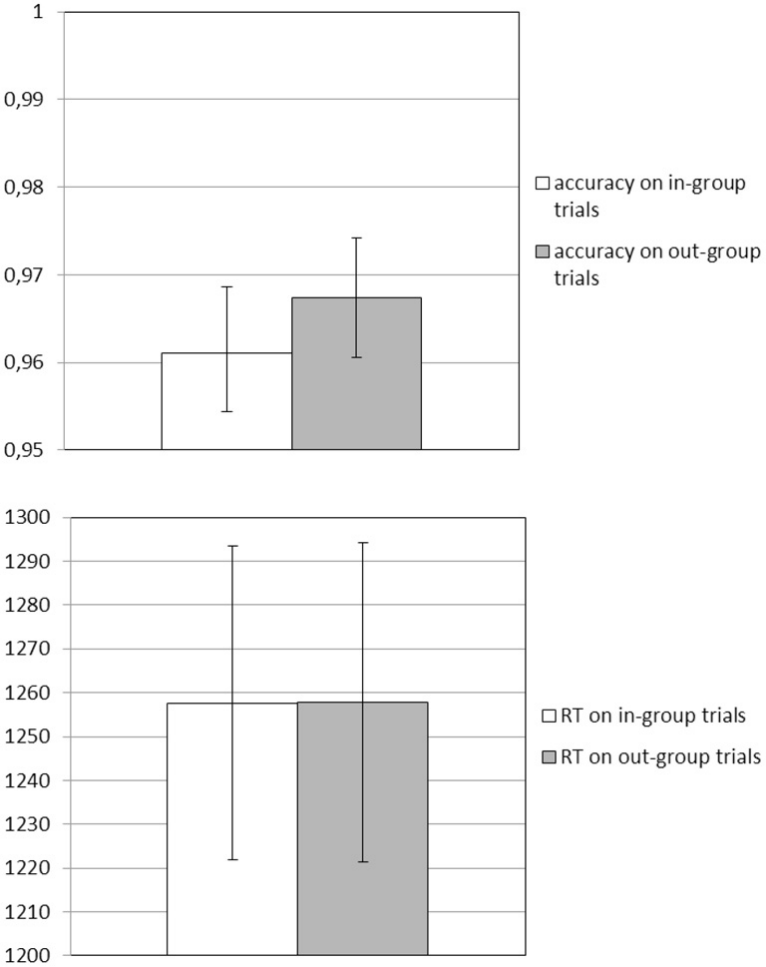
**Behavioral responses in the scanner: mean accuracy (top panel) and response times (RT; lower panel) while participants categorized in-group and out-group targets**.

In the scanner all participants were able to correctly categorize in-group and out-group faces (*M*_acc_ = 0.96, *SD* = 0.03; minimum accuracy = 0.90). There were no differences for accuracy and response latencies between the different stimulus types (*M*_accin−group_ = 0.96, *M*_accout−group_ = 0.97, *M*_rtin−group_ = 1257.6 ms, *M*_rtout−group_ = 1257.8 ms; all pairwise *t*-scores < 0.61, all *p* > 0.54, see Figure 3). Finally, identification was not related to how quickly or correctly participants responded to individual in-group and out-group targets (all *r*'s < 0.13, all *p* > 0.42).

### fMRI data

**Figure 4:**
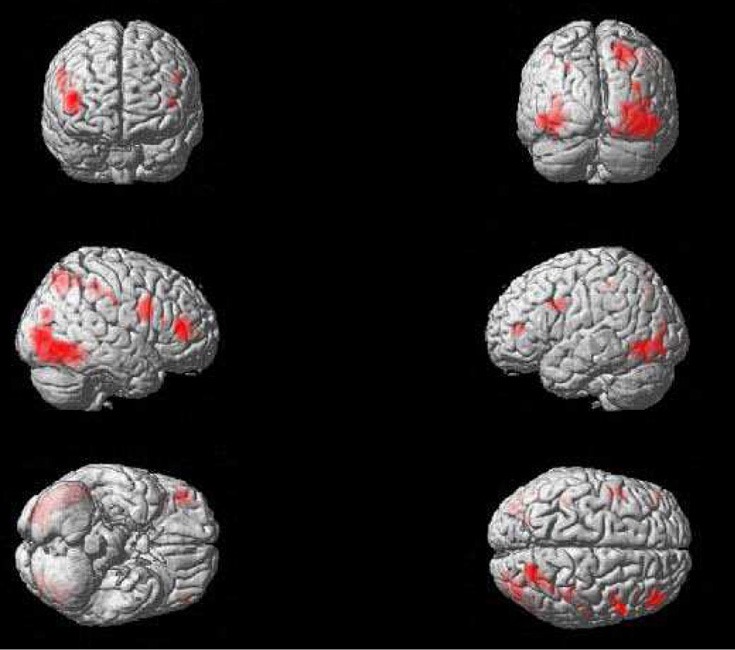
**Significant activations for self vs. familiar-other contrast, thresholded at *p* < 0.0005, with a contiguity threshold of 20 voxels**. Activations are shown on an individual brain rendered in 3D.

Replicating previous studies on self-face perception (Platek et al., 2008), the self—familiar other contrast revealed a bilateral, but right-dominated, pattern of activation in frontal, parietal and occipital regions (see Table 1 and Figure 4). This included strong activation in the regions that have been most frequently reported in fMRI research on self-face perception: the right inferior frontal gyrus and the right inferior and superior parietal lobule (Devue and Brédart, 2011).

**Table 1:** **Coordinates and peak activation statistics for clusters in self-familiar other contrast**.

**Region**	**Coordinates**				
	***k***	***x***	***y***	***z***	***T***
Right inferior, middle frontal gyrus	455	48	42	8	6.85
Right inferior frontal gyrus	467	50	8	22	5.38
Right inferior frontal gyrus	49	40	26	16	4.23
Left inferior, middle frontal gyrus	184	−50	4	32	4.77
Left inferior frontal gyrus	82	−46	46	8	4.29
Right parietal cortex including superior and inferior parietal lobule, precuneus	857	30	−62	50	4.65
Right postcentral gyrus	53	54	−24	40	4.21
Left inferior parietal lobule	43	−42	−38	48	4.00
Left precuneus	49	−24	−68	42	3.89
Right occipital and temporal cortex	2649	44	−64	−10	6.98
Left occipital and temporal cortex	1073	−34	−60	−4	5.74

*k, number of voxels in cluster. Coordinates (x, y, z) refer to Montreal neurological institute stereotaxic space (MNI)*.

The in-group—out-group X identification contrast yielded significant clusters of activation in the right inferior frontal gyrus, the right inferior and superior parietal lobule and left lingual gyrus (see Table 2): in all four areas, participants with higher identification scores showed increased activation to in-group members compared to out-group members. As noted above, these first three areas are the most frequently reported areas in fMRI research on self-face perception (Devue and Brédart, 2011).

**Table 2:** **Coordinates and peak activation statistics for clusters in in-group—out-group contrast, predicted by identification**.

**Region**	**Coordinates**				
	***k***	***x***	***y***	***z***	***T***
Right inferior frontal gyrus	21	46	8	40	4.05
Right inferior parietal lobule	73	40	−46	38	4.67
Right superior parietal lobule	40	30	−78	44	4.08
Left lingual gyrus	42	−12	−64	−6	4.20

*k, number of voxels in cluster. Coordinates (x, y, z) refer to Montreal neurological institute stereotaxic space (MNI)*.

**Figure 5:**
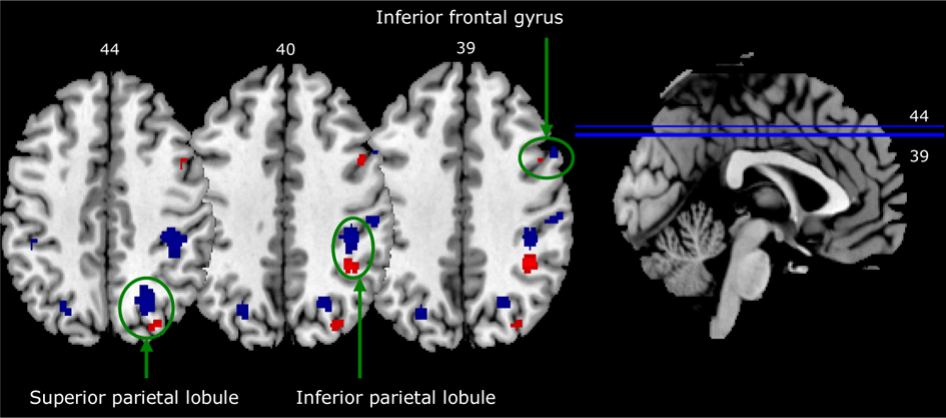
**Activation in closely adjacent areas in self—familiar contrast (blue) and in-group—out-group X identification contrast (red)**. Both contrasts are thresholded at *p* < 0.0005, with a contiguity threshold of 20 voxels. Images are in neurological format (right = right).

Figure 5 shows a striking pattern: three of the four areas activated in the in-group—out-group X identification contrast are closely adjacent to (but not overlapping with) activation clusters in the self—familiar other contrast. To statistically confirm this impression, we created spheres with an 8-mm radius around the activation peak in each of these clusters of activation in each of the in-group—out-group X identification contrasts, and then examined these spheres for voxels that were activated more by self than by familiar other.

This yielded significant clusters of activation in all three right-hemisphere regions: the right inferior frontal gyrus (*p*_cluster−corrected_ = 0.003; peak coordinates: 50, 8, 34; *t* = 4.50), the right inferior parietal lobule (*p*_cluster−corrected_ = 0.008; peak coordinates: 40, −40, 42; *t* = 3.71), and the right superior parietal lobule (*p*_cluster−corrected_ = 0.016; peak coordinates: 26, −72, 46; *t* = 3.72). A similar analysis for activation in the left lingual gyrus, not a typical self-relevant area, did not show any significant results.

We also examined the in-group—out-group contrast without the covariate of group identification. In line with our assumption that in-group/out-group distinctions are only meaningful to the degree that one identifies with the in-group, this did not yield any significant activation. Finally, we tested all of the reverse contrasts. The out-group—in-group contrast and the interaction with in-group identification did not reveal any significant activation. The familiar other—self contrast revealed significant clusters of activation in the right medial frontal gyrus, the right middle temporal gyrus, right precuneus, and the right fusiform gyrus (Table 3).

**Table 3:** **Coordinates and peak activation statistics for clusters in familiar other—self contrast**.

**Region**	**Coordinates**				
	***k***	***x***	***y***	***z***	***T***
Right medial frontal gyrus	33	4	54	−14	4.49
Right middle temporal gyrus	254	54	−16	−10	4.88
Right precuneus	353	6	−54	32	5.62
Right fusiform gyrus	103	54	−2	−30	5.20

*k, number of voxels in cluster. Coordinates (x, y, z) refer to Montreal neurological institute stereotaxic space (MNI)*.

## Discussion

The current research shows that people who identify highly with an in-group activate closely adjacent brain areas when they see faces of in-group members and when they see their own face. These closely adjacent brain areas for self and in-group were observed in the most frequently reported areas in previous research on self-face perception: the right inferior frontal gyrus and several regions in the right parietal cortex (i.e., the inferior parietal lobule and superior parietal lobule; see Platek et al., 2008; Devue and Brédart, 2011). It has been shown that these right parietal areas are crucial for making visual self-other distinctions, a primary factor in the development of *self*-awareness (Uddin et al., 2007). In our study, group identification was positively related to activation in these same areas when participants made in-group—out-group distinctions, which is the first step in *social* identity definition.

Because we used (previously) unfamiliar individuals as in-group and out-group stimuli, and randomized the group membership of these individuals between participants, the larger brain responses to in-group members than to out-group members cannot be explained in terms of familiarity or similarity in facial features. In addition, high and low identifiers did not differ in the number of trials needed to learn the in-group/out-group categorization, suggesting that differences in brain activation cannot be explained in terms of more/less exposure to the in-group and out-group faces, or other characteristics of the task. That is, although after some practice *all* participants were able to perform reasonably well on the categorization task, the neural activations underlying these categorizations differed in a meaningful way as a function of in-group identification.

A possible explanation for the activations we found in the right parietal cortex is that self-faces and in-group faces may have drawn more attention than faces of the familiar other and out-group members. Indeed, it has been demonstrated that the right parietal cortex is involved in attentional processes (Manly et al., 2003). This explanation would be perfectly in keeping with the notion that “attention to the self” forms an important component of self-consciousness (Decety and Sommerville, 2003). However, we are cautious to draw definitive conclusions about the role of attention in the current results as we did not observe significant differences in reaction times when categorizing in-group and out-group members.

An important finding is that although self-face perception and social identity yielded activation in closely adjacent brain areas, these areas did not overlap. In retrospect this may be explained by the notion that, in the end, self and in-group are different entities, even though they are based on partly similar processes. Nevertheless, the striking similarity in the pattern of activation raises the question whether the concepts of self and social identity are represented in a common domain of category-specific semantic organization (Mahon and Caramazza, 2009). That is, representations of self and in-group may share an evolutionarily relevant history (Brewer, 2007), which has been proposed as an important basis for conceptual organization in the brain (Mahon and Caramazza, 2009).

We did not find any significant activation in the in-group—out-group contrast without inclusion of in-group identification as a covariate. This is in keeping with our assumption that the perception of in-group members should only lead to social-identity-relevant brain activation for those who see the group as an important part of their self-concept or identity. At the same time, this finding may seem at odds with previous research showing brain activation in an in-group—out-group contrast without controlling for identification (e.g., Hart et al., 2000; Van Bavel et al., 2008; Wright et al., 2008; Cikara et al., 2011). However, these previous studies were either conducted in the context of an inter-group competition (Van Bavel et al., 2008; Cikara et al., 2011), which typically increases in-group identification, or involved social categories that are directly visible and therefore more or less chronically salient (e.g., racial, age or gender groups; Hart et al., 2000; Wright et al., 2008). In the current non-competitive and rather “minimal” inter-group context, using previously unfamiliar in-group members as stimuli, the absence of activation in the in-group—out-group contrast without controlling for identification is perhaps less surprising.

Although social categorization (i.e., making the distinction between “us” and “them”), forms the basis of social identity, we do not want to suggest that the brain activation associated with this process can fully capture such a complex phenomenon as “social identity.” As is the case for the personal self-concept (e.g., self-reflection, self-esteem), social identity has different aspects, which most likely involve different, bilateral, neural networks (Lieberman, 2007; Uddin et al., 2007). In relation to this, social identity can be made salient by exemplars of the in-group, as in the current research, but also by traits, group names, symbols, etc. (Morrison et al., 2012). At a more abstract level, apart from face perception, (social) identity can also be developed and activated through other modalities such as words or traits related to certain groups, sound (speech with a certain accent) or even smell (Coppin et al., under review). Nonetheless, visual face-perception is an important source for early social categorization processes in day-to-day life and can set in motion important psychological processes, such as stereotypes and social identity, that in turn affect human interactions.

Finally, it is important to note that in the current work we solely focused on a basic cognitive component of social identity, namely in-group face perception. However, social-categorization—and the social identity it provides—is closely intertwined with emotion and motivation (Tajfel and Turner, 1979). Indeed, previous research has shed light on how the neural reward system responds to in-group success and out-group failure (Cikara et al., 2011), and how social categorization relates to the neural correlates of in-group bias (Van Bavel et al., 2008). Rather than examining the consequences of social identity, we examined social identity in its most basic cognitive sense, in terms of in-group—out-group distinctions, and drew a parallel with the personal self-concept in its most basic sense, in terms of self—other distinctions (Uddin et al., 2007). Future research should examine how the different aspects of social identity relate to different networks in the brain, and how these networks interactively shape inter-group behavior (e.g., discrimination), and related important outcome variables such as collective self-esteem.

### Conflict of interest statement

The authors declare that the research was conducted in the absence of any commercial or financial relationships that could be construed as a potential conflict of interest.

## Footnotes

^1^In order to isolate activation associated with self-face perception, and to exclude any activation associated with social identity, we created a contrast between self and a familiar other who shared the social identity that was relevant for the ingroup-outgroup contrast (university affiliation). That is, subtracting activation related to social identity in the self-familiar other contrast yields the purest activation for self-face perception.
